# Cerium End-Deposited Gold Nanorods-Based Photoimmunotherapy for Boosting Tumor Immunogenicity

**DOI:** 10.3390/pharmaceutics15041309

**Published:** 2023-04-21

**Authors:** Yanlin Feng, Yumei Xu, Zhaoyang Wen, Xin Ning, Jianlin Wang, Deping Wang, Jimin Cao, Xin Zhou

**Affiliations:** 1 Key Laboratory of Cellular Physiology, Ministry of Education, the Department of Physiology, Shanxi Medical University, Taiyuan 030001, China; 2 Department of Medical Imaging, Shanxi Medical University, Taiyuan 030001, China

**Keywords:** cerium end-deposited gold nanorods, photoimmunotherapy, PD-1 blockade, triple-negative breast cancer

## Abstract

Background: Triple-negative breast cancer (TNBC) was closely related to high metastatic risk and mortality and has not yet found a targeted receptor for targeted therapy. Cancer immunotherapy, especially photoimmunotherapy, shows promising potential in TNBC treatment because of great spatiotemporal controllability and non-trauma. However, the therapeutic effectiveness was limited by insufficient tumor antigen generation and the immunosuppressive microenvironment. Methods: We report on the design of cerium oxide (CeO_2_) end-deposited gold nanorods (CEG) to achieve excellent near-infrared photoimmunotherapy. CEG was synthesized through hydrolyzing of ceria precursor (cerium acetate, Ce(AC)_3_) on the surface of Au nanorods (NRs) for cancer therapy. The therapeutic response was first verified in murine mammary carcinoma (4T1) cells and then monitored by analysis of the anti-tumor effect in xenograft mouse models. Results: Under near-infrared (NIR) light irradiation, CEG can efficiently generate hot electrons and avoid hot-electron recombination to release heat and form reactive oxygen species (ROS), triggering immunogenic cell death (ICD) and activating part of the immune response. Simultaneously, combining with PD-1 antibody could further enhance cytotoxic T lymphocyte infiltration. Conclusions: Compared with CBG NRs, CEG NRs showed strong photothermal and photodynamic effects to destroy tumors and activate a part of the immune response. Combining with PD-1 antibody could reverse the immunosuppressive microenvironment and thoroughly activate the immune response. This platform demonstrates the superiority of combination therapy of photoimmunotherapy and PD-1 blockade in TNBC therapy.

## 1. Introduction

Triple-negative breast cancer (TNBC) is generally considered a poor prognosis with high recurrence and mortality because of no targeted receptors found and lack of effective treatment [1,2]. As a result, cancer immunotherapy, which uses the patient’s own immune system to attack cancer cells, has thus attracted researchers’ wide attention and achieved therapeutic efficacy in clinical trials [3,4]. However, immunotherapy was only beneficial to a small percentage of patients due to inadequate activation of immune systems and subsequent adverse effects [5,6]. Many efforts have been put forth by combining diverse treatments with immunotherapy for TNBC to enhance immune responses, including chemotherapy [7], radiotherapy [8,9], photothermal therapy (PTT), and photodynamic therapy (PDT) [10,11,12,13]. Nevertheless, there are severe side effects associated with chemotherapy and radiotherapy in clinics. Photoimmunotherapy, composed of photothermal/photodynamic immunotherapy, has attracted great attention in inducing and promoting immune responses against tumors with unique advantages such as low cost, noninvasive properties, and excellent spatiotemporal controllability [14,15,16]. Specifically, under light, irradiation, PTT and PDT agents could trigger cell damage through local hyperthermia and the production of reactive oxygen species (ROS). During the process, the dying tumor cells released damage-associated molecular patterns (DAMPs), which subsequently promoted dendritic cells (DCs) to perform their antigen-presenting function, finally resulting in immunogenic cell death (ICD) [17,18,19,20]. Moreover, to induce prominent ICD better and enhance tissue penetration, the light absorption of phototherapy agents was adjusted to a near-infrared (NIR) region [21]. Although promising, their access to actual therapy is restricted by inadequate activation of the systematic immune response as insufficient tumor antigen production and the immunosuppressive microenvironment. Therefore, it is an urgent need to create an effective photoimmune agent that can produce a sufficient tumor antigen to effectively activate innate immunity for the treatments of TNBCs.

A plasma meta catalyst has been widely used as a phototherapy platform to increase phototherapeutic efficacy due to its unique electronic properties. Upon resonant light excitation, the localized surface plasmon resonance (LSPR) can create field enhancements near the particle to generate charge carriers [22,23]. These abundant charges undergo chemical and energy transformations to generate abundant ROS [24,25,26,27] and release vast heat through the electron-phonon relaxation process [28,29]. Among various plasma meta-catalysts, gold nanoparticles have attracted a lot of attention due to their strong LSPR, well-controlled surface chemistry, and ideal biocompatibility [30,31,32,33]. However, the ultrafast recombination of electron–hole pairs restrict the utilization efficiency of plasmon-induced hot carriers. Fortunately, the Schottky barrier could be formed after the combination of semiconducting nanomaterials with metal, and the hot electrons from metal could transfer to the conduction band of semiconductors, thus boosting the production of hot electrons, which can result in improved PTT and PDT performance [14,22,34,35].

**Figure 1:**
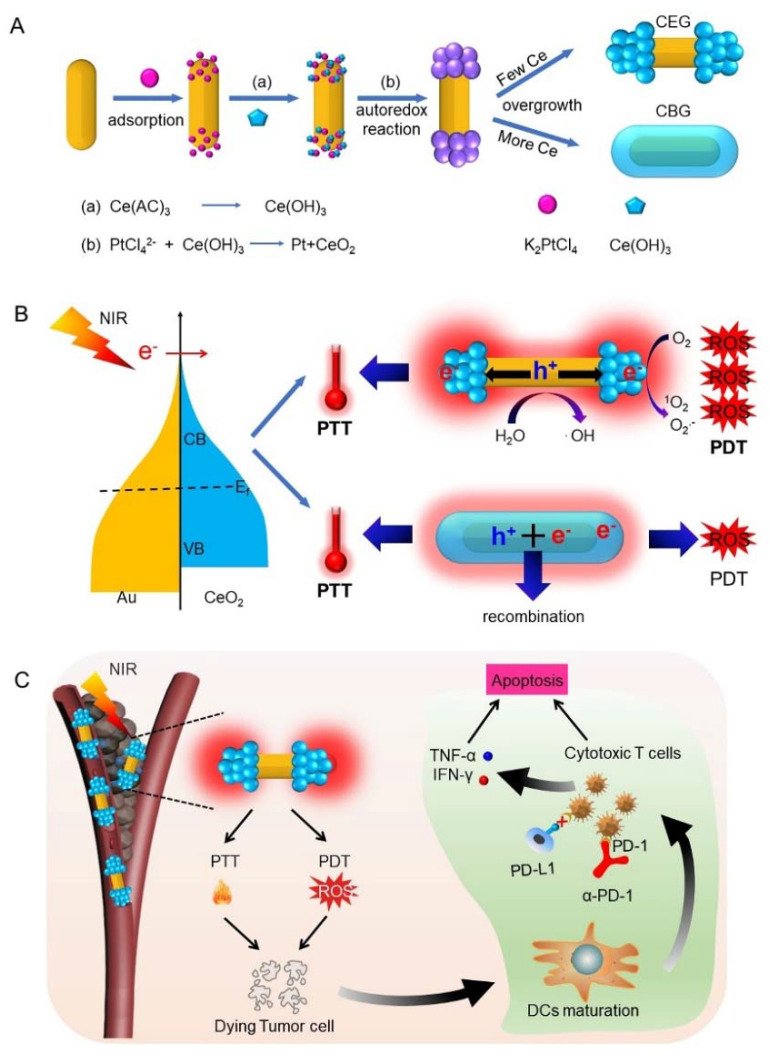
Schematic illustration of the combination therapy of photoimmunotherapy synergizes with PD-1 blockade in triple-negative breast cancer using CEG NRs. (**A**) Illustration of the synthesis process of CEG and CBG [14]. (**B**) NIR laser-activated charge carrier spatial separation to release heat and promote more ROS production for CEG than CBG. (**C**) CEG NRs displayed significant PDT and PTT effects to activate systemic immunity to destroy tumor cells together with α-PD-1 after intravenous administration to breast cancer-bearing mice.

In this study, to achieve a more significant immune response to phototherapy, biocompatible cerium oxide (CeO_2_)-end-deposited gold (CEG) nanorods (NRs) (Figure 1A) with excellent plasmonic properties were synthesized for TNBCs therapy. CeO_2_ was selected as a candidate for plasmonic photocatalysis because it is an n-type semiconductor and can form a Schottky barrier with gold nanorods (GNRs) to facilitate the hot-electron separation [36,37]. With 808 nm laser irradiation, electron–hole spatial separation occurred easily along the longitudinal axis of GNRs, generating more significant hot electrons in comparison with CeO_2_ body-deposited gold nanorods (CBG), which suffer from fast recombination because of homogeneous coating. The efficient hot-electron generation could release heat based on electron–phonon relaxation and generate ROS through energy and chemical transformation processes [24,38], affording outstanding PTT and PDT performance to destroy tumor cells under light (Figure 1B). In the meantime, dying tumor cells after phototherapy can release tumor-associated antigens (TAAs), which play the role of an in situ cancer vaccine to promote DCs maturation, in turn leading to a high degree of CD8^+^ T cells infiltration and an increased secretion of pro-inflammatory cytokines. To comprehensively activate the immune responsive, program death-1 (PD-1) antibody (α-PD-1) was further adopted to prevent the tumoral immunosuppression and raise the number of cytotoxic T cells, which produce various cytokines in serum, enhancing the cancer immunotherapy (Figure 1C).

## 2. Materials and Methods

### 2.1. Materials

Cetyltrimethylammonium bromide (CTAB), Cerium (III) acetate hydrate (Ce (AC)_3_ × H_2_O), 2′, -7′-dichlorodihydrofluorescein diacetate (DCFH-DA), terephthalic acid (TA), singlet oxygen sensor green (SOSG), MitoSox Red and JC-1 were obtained from Sigma. Sodium borohydride (NaBH_4_), ascorbic acid (AA), dimethylaminopropyl-3-ethylcarbodiimide hydrochloride (EDC), N-hydroxysuccinimide (NHS) and Cyanine 7 (Cy7) were purchased from Aladdin. Gold chloride trihydrate (HAuCl_4_·3H_2_O) and potassium tetrachloroplatinate (I) (K_2_PtCl_4_) were purchased from ACMEC. Hydrochloric acid (HCl) and silver nitrate (AgNO_3_) were obtained from Beijing Chemical Reagent Company (Beijing, China). Thiol-terminated PEG (PEG-SH, Mw = 5000) and thiol PEG amine (SH-PEG-NH_2_) were supplied by Ponsure (Shanghai, China). Roswell Park Memorial Institute (RPMI) 1640 medium, phosphate buffer (PBS), and fetal bovine serum (FBS) were all purchased from Procell. A Cell Counting Kit-8 (CCK-8) was acquired from APE × BIO. A Superoxide anion Content Detection Kit was obtained from Solarbio. Celcein-AM, PI, One Step terminal deoxynucleotidyl transferase dUTP nick-end labeling (TUNEL) Apoptosis Assay Kit and Annexin V-FITC Apoptosis Detection Kit were purchased from Beyotime. α-PD-1 was acquired from Bio X Cell. Tumor necrosis factor α (TNF-α), interferon γ (IFN-γ), and immune globulin G (IgG) were purchased from BD Biosciences. Anti-CD86 PE, anti-CD80 APC, FITC anti-mouse CD3 antibody, and APC anti-mouse CD8 antibody were acquired from Biolegend. All of these materials were used directly without further purification.

### 2.2. Characterization

The morphology was characterized by transmission electron microscopy (TEM) and scanning transmission electron microscopy (STEM)-high-angle annular dark field (HAADF) (FEI Tecnai G20). Using a Rigaku-Dmax 2500 diffractometer, we recorded X-ray diffraction patterns (XRD). The UV-Vis spectra were measured using a UV-Vis/visible/NIR spectrometer (Hitachi UH5700). Using the Malvern Nanosizer ZS, the zeta potential and dynamic light scattering (DLS) were measured.

### 2.3. Synthesis of Gold Nanorods (GNRs)

For the synthesis of GNRs, the seed-mediated growth method was used [39]. Iced NaBH_4_ solution (10 mM, 600 µL) was added into the mixture containing HAuCl_4_ (10 mM, 250 µL) and CTAB (0.1 M, 9.75 mL) and aged at 30 °C for 2 h. Then, HAuCl_4_ (10 mM, 10 mL), AgNO_3_ (10 mM, 2 mL), HCl (1 M, 4 mL), and AA solution (0.1 M, 1.6 mL) were successively added into the CTAB solution (0.1 M, 200 mL) under stirring. Following stirring for 2 min, the prepared seed solution (50 µL) was quickly injected and let stand at 30 °C. After overnight growth, it was centrifugated and washed with water at 7000 rpm for 10 min. The as-prepared GNRs were washed twice in pure water and then redispersed in water.

### 2.4. Synthesis of CEG and CBG NRs

CEG and CBG NRs were prepared based on the literature with slight modifications [14]. For CEG NRs, the above GNRs were centrifugated and redispersed in CTAB solution (0.1 mM, 25 mL). Then, K_2_PtCl_4_ solution (0.5 mM, 1 mL), Ce(AC)_3_ solution (50 mM, 2.5 mL), and H_2_O (21.5 mL) were added in an oven at 100 °C for 1 h to obtain the CEG NRs. Through centrifugation and washes twice with pure water, the product was obtained. CBG NRs were prepared in a similar way, except that the concentrations of K_2_PtCl_4_ solution and Ce(AC)_3_ solution were 2 mM and 0.2 M, respectively.

### 2.5. Surface Modification of CEG NRs

mPEG-SH solution (5 mg/mL, 10 mL) aqueous solution was added into 250 mL CEG NRs (optical density (OD) = 1) solution and stirred in the dark for 48 h at room temperature. Excess PEG was removed from the sample by centrifuging at 10,000 rpm for 10 min and washing with pure water several times.

### 2.6. Cy-7 Labeling of CEG NRs

CEG NRs were firstly functionalized by SH-PEG-NH_2_ (Mw = 5 kD) for further binding to the carboxyl groups of Cy-7. In detail, 20 mL SH-PEG-NH_2_ aqueous solution (500 µg/mL) was added to 20 mL CEG NRs aqueous suspension (OD = 10) dropwise. After overnight stirring in the dark, the above mixture was centrifuged at 7000 rpm and then washed with water several times and dispersed in 20 mL pure water, named CEG-NH_2_. At the same time, 1 mL of NHS (2 mg/mL) and 1 mL of EDC (2.4 mg/mL) were infused with 1 mL of Cy-7 aqueous solution (1 m g/mL) at stirring for 24 h under dark conditions. After being concentrated and washed with water three times, the above CEG-NH_2_ aqueous solution was added, and the mixture was stirred continuously for 24 h. The yield Cy-7 labeled CEG NRs were obtained by centrifuging and then washed thrice in water.

### 2.7. Photothermal Test and Infrared Thermal Image of GNRs, CEG, and CBG NRs

1 mL of GNRs, CEG, and CBG NRs with different concentrations (OD = 2, 1, and 0.5) were respectively placed into quartz cuvettes and irradiated with a 808 nm laser (1 W/cm^2^) for 10 min. The temperature elevation of the suspension was measured every 30 s. Using an infrared thermal imager (Fotric 1204), infrared thermal images were taken every two minutes.

### 2.8. Photodynamic Performance of GNRs, CEG, and CBG NRs

Total ROS, singlet oxygen (^1^O_2_), hydroxyl radical (^•^OH), and superoxide anion ($O_{2}^{•-}$) generation of GNRs, CEG, and CBG NRs were determined by DCF [40], SOSG [41], TA [42], and XTT [43], respectively. DCF is prepared by hydrolysis of DCFH-DA. Briefly, 80 µL DCF (29 µM), SOSG (12 µM), TA (10 µM), and XTT (100 µM) were infused with 20 µL GNRs, CEG, and CBG NRs with different concentrations (OD = 2, 1, and 0.5). After 24 h of incubation, a 808 nm laser (1 W/cm^2^) was used to irradiate the above mixture for 10 min. DCF, SOSG, or TA fluorescence emission spectra, were measured with an excitation wavelength of 490, 394, and 315 nm, respectively. Furthermore, absorbance spectra of XTT were recorded across the range of 410 to 550 nm.

### 2.9. Cell Culture

Mouse breast cancer 4T1 cells were acquired from the Shanghai Institute of Biological Sciences and cultured in T-25 flasks with RPMI-1640 medium at 5% CO_2_ and 37 °C. We replaced the medium every two days with a fresh medium. The attached cells were removed from the T-25 flasks for passage by trypsinization.

### 2.10. Cell Viability Assessments by CCK-8 and Live/Dead Staining

The viability of 4T1 cells was assessed by CCK8 and live/dead staining assays. About 1 × 10^4^ 4T1 cells were dispersed into 100 µL of fresh culture medium and inoculated into each well of the 96-well plate and incubated for 24 h. Then, we removed the culture medium and treated the cells with 100 µL of fresh medium containing different concentrations of CEG NRs (OD = 0, 0.25, 0.5, 1, and 2) for 6 h of incubation. Then, the cells were irradiated with a 808 nm laser (1 W/cm^2^) for 10 min and continued to be incubated for 18 h. The cells in the control group were cultured at 37 °C in the dark. After finishing the incubation, 10 µL of CCK8 reagent was added to each well and then incubated for another 2–4 h. The absorbance of the solution was measured at 450 nm by a microplate reader. As to live/dead cell staining, following a wash with PBS, the cells were incubated with calcein AM (2 µM) and propidium iodide (PI, 4 µM) at 37 °C for 30 min. Then the viability of 4T1 cells was visualized using an Olympus fluoresce microscope.

### 2.11. Cellular ROS Detection

Flow cytometry and fluorescence microscopy were used to detect ROS levels with H2DCFDA. About 1.5 × 10^5^ 4T1 cells were dispersed into 1.5 mL of fresh culture medium and inoculated into each well of the 6-well plate, and incubated for 24 h. On the second day, following the removal of the culture medium, the cells were incubated with a 1.6 mL fresh medium containing various concentrations of CEG NRs (OD = 0, 0.25, 0.5, 1, and 2). For the dark group, cells were cultured continuously in the dark; for the NIR laser-triggered phototherapy group, the cells were irradiated with a 808 nm laser (1 W/cm^2^) for 10 min after 6 h of incubation and continued to be cultured for 18 h. Once the incubation process was completed on the third day, H2DCFDA (10 µM) was added to each well and then incubated for another 20 min. For the flow cytometry assay, cells were isolated by trypsin and analyzed using the Beckman Coulter FC500 flow cytometer after two washes with PBS. For fluorescence microscope imaging, the cells were photographed using an Olympus fluorescence microscope after washing the cells with PBS.

### 2.12. Analysis of Mitochondrial Membrane Potential and Superoxide Generation

JC-1 and Mitosox Red probes were used to measure the mitochondrial membrane potential and superoxide generation. On the first day, 1.5 × 10^5^ 4T1 cells were dispersed into 1.5 mL of culture medium and inoculated into each well of the 6-well plate, and cultured for 24 h. On the second day, after the removal of the culture medium, the cells were incubated with a 1.6 mL fresh medium containing various concentrations of CEG NRs (OD = 0, 0.25, 0.5, 1, and 2). For the dark group, cells were cultured continuously for 24 h; for the NIR light-triggered phototherapy group, the cells were irradiated with a 808 nm laser (1 W/cm^2^) for 10 min after 6 h of incubation and continued to be incubated for 18 h. On the third day, after finishing the incubation, JC-1 (5 µM) or MitoSox Red (5 µM) was added to each well and then incubated for 20 min. Finally, fluorescence photographs were obtained using an Olympus fluorescence microscope.

### 2.13. HSP-70 and HO-1 Expression Analyzed by Western Blot

About 1.5 × 10^5^ 4T1 cells were dispersed into 1.5 mL of culture medium and inoculated into each well of the 6-well plate, and cultured for 24 h. On the second day, after the removal of the culture medium, the cells were incubated with a 1.6 mL fresh medium containing various concentrations of CEG NRs (OD = 0, 0.25, 0.5, 1, and 2). For the dark group, cells were incubated continuously for 24 h; for the NIR light-triggered phototherapy group, the cells were incubated with CEG NRs for 6 h and then irradiated with a 808 nm laser (1 W/cm^2^) for 10 min, followed by 18 h of incubation. On the third day, after finishing the incubation, wash the cells with PBS three times. A 20 μL lysis buffer was added to each well to collect the cells for protein content quantified to 20 μg using the Bradford method for electrophoretic analysis at medium 80 V and then transferred to the PVDA membrane. Following blocking the PVDA membrane with 10% skim milk for 2 h, the PVDA membrane was incubated with β-actin (1:1000, Beyotime), heat shock protein 70 (HSP-70) antibody (1:1000, Abcam) or Heme oxygenase-1 (HO-1) monoclonal antibody(1:1000, Abcam) for 12 h at 4 °C. Then the membrane was washed with TBS/T solution for three times and then incubated with goat anti-mouse horseradish peroxidase-conjugated secondary antibody (1:1000, Beyotime) for 1 h at room temperature. After three washes with TBS/T solution, a Bio-Rad imaging system was used to visualize the membrane.

### 2.14. Cell Apoptosis Test by Flow Cytometry

On the first day, 1.5 × 10^5^ 4T1 cells were dispersed into 1.5 mL of culture medium and inoculated into each well of the 6-well plate, and incubated for 24 h. On the second day, after the removal of the culture medium, the cells were incubated with a 1.6 mL fresh medium containing various concentrations of CEG NRs (OD = 0, 0.25, 0.5, 1, and 2). For the dark group, the cells were cultured continuously for 24 h; for the NIR light-triggered phototherapy group, the cells were irradiated with a 808 nm laser (1 W/cm^2^) for 10 min after 6 h of incubation and continued to be incubated for 18 h. On the third day, the cells were digested by trypsin and incubated with Annexin V-FITC/PI for flow cytometer analysis.

### 2.15. In Vivo Fluorescence Imaging for Biodistribution Analysis

When the tumors reached about 100 mm^3^, CEG-Cy-7 NRs (OD = 60, 100 μL) were administered intravenously to the tumor-bearing mice. After 1, 12, and 24 h following the injection, in vivo fluorescence imaging was conducted in anesthetized mice (Maestro In Vivo Imaging System (CRi, MA)).

### 2.16. In Vivo Infrared Thermal Imaging

When the tumors reached about 100 mm^3^, CEG NRs (OD = 60, 100 μL) were administered intravenously to the mice. After 24 h injection, the tumor site of the mice was irradiated with a 808 nm laser (1 W/cm^2^) for 10 min. Infrared thermal images were taken every 2 min under NIR light with Fotric 1204.

### 2.17. In Vivo Phototherapeutic Evaluation

Female BALB/c mice (6 to 8 weeks old, 17 to 19 g, purchased from Beijing Vital River) were used to establish the xenograft mouse model. In animal experiments, all animal operating procedures are in accordance with the standards approved by the Animal Research Ethics Committee of the Shanxi Medical University and the guidelines for the Care and Use of Laboratory Animals (NIH, revised 2011) with the approval number SYDL2019012. A total of 10^7^ 4T1 cells were dispersed in 50 µL PBS and inoculated subcutaneously on the back of mice. When the tumors reached about 100 mm^3^, the 4T1 tumor-bearing mice were divided into six groups in a random assortment model (four mice in each group) for different treatments: (1) PBS; (2) CEG; (3) NIR; (4) α-PD-1; (5) CEG + NIR; (6) CEG + NIR + α-PD-1. Then, 100 µL of PBS or CEG (OD = 60) was intravenously injected into the tumor-bearing mice. For groups (3), (5), and (6), the tumors were irradiated with a 808 nm laser (1 W/cm^2^) for 10 min after 24 h injection. For groups (4) and (6), the mice were injected with α-PD-1 antibody 50 µg per mouse on days 1, 4, 7, and 10. Tumor volume and body weight were measured daily. All mice were sacrificed 14 days after treatment. Major organs (heart, liver, spleen, lung, kidney) and tumors were collected, embedded, and sectioned. Hematoxylin and Eosin (H&E) staining and immunostaining of the sections were performed. Additionally, the expression of IgG, TNF-α, and IFN-γ was analyzed by collecting the serum of six groups of mice according to the specifications.

### 2.18. Immunohistochemistry Analysis

Tumors were sectioned in accordance with the standardized experimental procedures. The 4 μm thick tumor sections were incubated with CD3 or CD8 antibody and corresponding HRP combined with secondary antibody. Finally, tumor sections were observed under a microscope.

### 2.19. Immunofluorescence Assay for T Cells

Tumors were sectioned according to the standardized experimental procedures [44]. The 4 μm thick tumor sections were incubated with CD3 or CD8 antibody and dye-conjugated secondary antibody. Finally, an anti-fluorescence quenching agent containing DAPI was dropped onto tumor sections, and an Olympus fluorescence microscopy was used to examine the tumor sections.

### 2.20. Statistical Analysis

Quantitative data were expressed as mean ± standard deviation (S.D.). Statistical comparisons were made by ANOVA analysis. The *p* value < 0.05 was regarded as a significant difference.

## 3. Results and Discussion

### 3.1. Preparation and Characterization of CEG and CBG NRs

**Figure 2:**
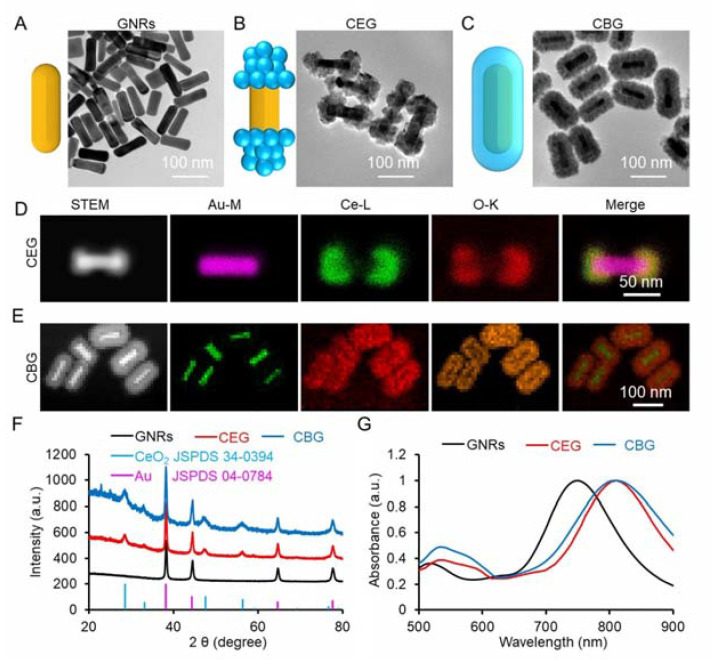
Physicochemical characterization of CEG and CBG NRs. (**A**–**C**) TEM images of GNRs, CEG, and CBG NRs, respectively. (**D**,**E**) STEM and EDS elemental mapping images of CEG and CBG NRs. (**F**) The XRD pattern of CEG and CBG NRs. (**G**) The UV-Vis-NIR absorption spectra of GNRs, CEG, and CBG.

The synthesis process for CEG and CBG NRs is shown in Figure 1A, which was successively prepared through several steps. Firstly, GNRs were synthesized via a seed-mediated method [45,46], as observed by TEM in Figure 2A. Then K_2_PtCl_4_ was added, which was preferentially adsorbed on the two ends of GNRs. As a result, the ceria precursor (cerium acetate, Ce(AC)_3_) that is subsequently added can be hydrolyzed into Ce(OH)_3_ and oxidized to CeO_2_ by potassium tetrachloroplatinate (K_2_PtCl_4_) at two ends of GNRs when the temperature is 100 °C [47]. Whereafter, the CeO_2_ further grew to produce CEG or CBG NRs when a different dose of K_2_PtCl_4_ and Ce(AC)_3_ was added (Figure 2B,C). The structure, chemical formation, and elemental distribution of CEG and CBG NRs were further confirmed by STEM with energy-dispersed X-ray spectroscopy (EDS) elemental mapping and high-resolution transmission electron microscopy (HRTEM) images. It could be observed that Ce and O elements were located at the two ends of GNRs for CEG NRs (Figure 2D) while uniformly surrounding the outside of GNRs for CBG NRs (Figure 2E). EDX spectrum (Figure S1) also verified the signals of Au, Ce, and O. The weight percentage (wt%) and atomic weight percentage (At %) values of all elements in CEG and CBG were listed in Tables S1 and S2. HRTEM images revealed that the lattice fringes of both CEG or CBG NRs matched well with the crystalline properties of Au rod and CeO_2_ (Figure S2). The XRD patterns also displayed the diffraction peaks of Au and CeO_2_ (Figure 2F), further proving the successful synthesis of CEG or CBG NRs. To ensure the potential photothermal/photodynamic performance, the absorbance features of CEG or CBG NRs were further investigated. UV-Vis-NIR absorption spectra showed that CeO_2_ coating could redshift the LSPR peak of GNRs from 750 to 810 nm for both CEG and CBG NRs (Figure 2G). To better understand the advantage of CEG NRs, the LSPR peak was finely adjusted as closely as possible to the laser wavelength, and the optical density (OD) values were adjusted to 1.0.

### 3.2. Photothermal and Photodynamic Performance of CEG and CBG NRs

**Figure 3:**
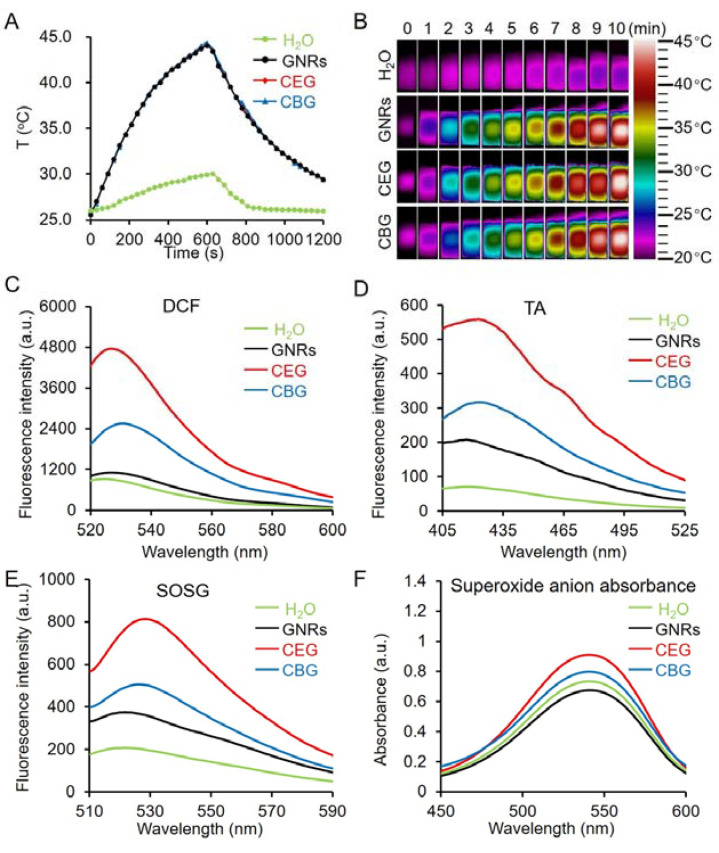
The photothermal and photodynamic performance of GNRs, CEG, and CBG NRs. (**A**) Heating and cooling curves and (**B**) infrared thermal images of GNRs, CEG, CBG NRs, and H_2_O. Total ROS, abiotic ^•^OH, ^1^O_2_, and O_2_^•−^ assessments of GNRs, CEG, and CBG NRs (OD = 1) upon 808 nm laser irradiation (1 W/cm^2^, 10 min) assessed by DCF (**C**), TA (**D**), SOSG (**E**) and superoxide anion assay (**F**), respectively.

The photothermal performance of GNRs, CEG, and CBG NRs was investigated by tracking the temperature elevation upon a 808 nm laser irradiation at the power density of 1 W/cm^2^ for 10 min by using a thermometer and thermal infrared imaging. Figure 3A,B showed that GNRs, CEG, and CBG NRs could induce similar temperature elevation to 44, 44.1, and 44.2 °C at the same OD (OD = 1.0), and the heating and cooling curves of GNRs, CEG, and CBG are almost perfectly matched and stacked together. Under the same conditions, the water temperature only raised by 4 °C. The photothermal conversion efficiency (ƞ) of all three is almost the same, which were calculated to be 34.1, 34.7, and 34.9% in accordance with Roper’s method [48] for GNRs, CEG, and CBG NRs (Figure S3), coinciding with their temperature elevation curves.

The photodynamic performance upon 808 nm laser irradiation (1 W/cm^2^, 10 min) was evaluated through the measurement of ROS production. Firstly, the DCF assay was utilized to detect the ability of the total generation of ROS. Figure 3C showed that CEG exhibited the most significant DCF fluorescence enhancement upon a 808 nm laser irradiation, followed by CBG and GNRs, implying that CEG has the greatest capacity to produce ROS. Instead, they could not generate ROS without NIR laser irradiation (Figure S4A). To further identify the ROS types, specific fluorescent probes, TA, SOSG, and Micro Superoxide Anion Assay Kit were employed to detect ^•^OH, ^1^O_2,_ and O_2_^•−^ generation. Figure 3D–F demonstrated that CEG could induce the highest TA and SOSG fluorescence intensity and superoxide anion absorbance intensity, followed by CBG and GNRs, certifying the fact that CEG holds the maximum ROS generation ability, including ^•^OH, ^1^O_2,_ and O_2_^•−^, followed by CBG and GNRs. Similarly, no ^•^OH, ^1^O_2,_ or O_2_^•−^ can be produced without NIR irradiation (Figure S4B–D). Notably, coupling CeO_2_ with GNRs (CEG and CBG NRs) generated more ROS because of the facilitated hot-electron separation, and CEG possessed more effective electron–hole separation to produce more ROS. As a result, CEG was chosen for the subsequent in vitro and in vivo studies. Before that, photostability was studied. Figure S5 illustrated that after four cycles of 808 nm laser irradiation (1 W/cm^2^, 10 min), the absorption profile (Figure S5A), the temperature elevation curve (Figure S5B), and the morphology of CEG (Figure S5C) were almost unchanged, indicating the excellent stability of CEG under 808 nm laser irradiation. Moreover, in order to enhance biocompatibility for possible applications in the biomedical field, the surface of CEG was further conjugated with thiol-terminated PEG (PEG-SH, Mw = 5000), as confirmed by zeta potentials and hydrodynamic sizes (Figure S6). The following experiments were conducted using PEG-conjugated CEG both in vitro and in vivo.

### 3.3. In Vitro Phototherapeutic Effect of CEG

**Figure 4:**
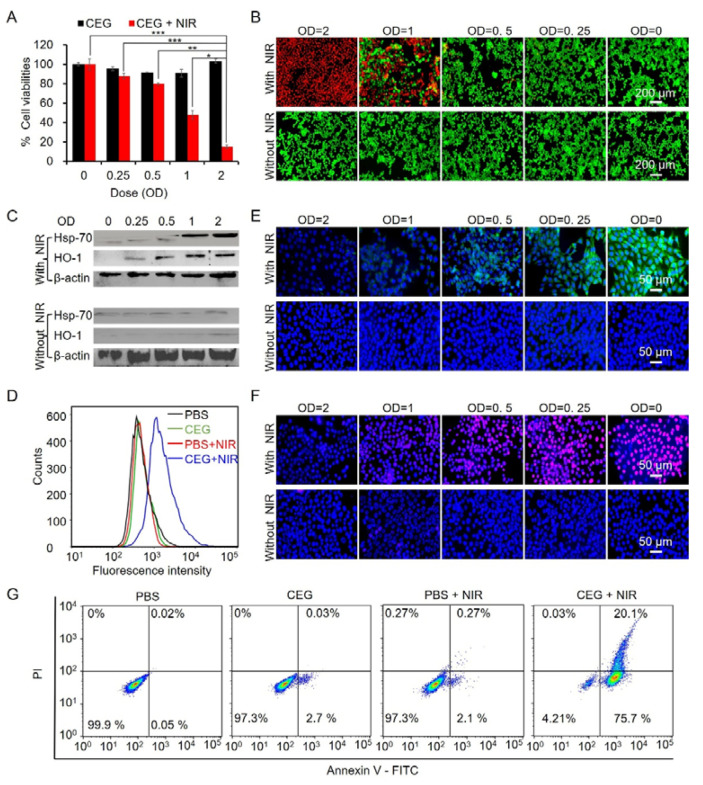
In vitro phototherapeutic efficacy of CEG in 4T1 cells. (**A**) Result of CCK-8 assay showing the cell viability of 4T1 cells following the treatment of CEG (OD = 0, 0.25, 0.5, 1, and 2) without or with 808 nm laser irradiation (1 W/cm^2^, 10 min). (**B**) Live/dead staining of 4T1 cells treated with CEG (OD = 0, 0.25, 0.5, 1, and 2) without or with 808 nm laser irradiation (1 W/cm^2^, 10 min). (**C**) Results of western blotting showing the expression levels of HSP-70 and HO-1 without or with NIR laser irradiation. (**D**) Flow cytometric analysis of intracellular ROS levels in 4T1 cells after the treatment of CEG upon 808 nm laser irradiation or not. CLSM images of (**E**) mitochondrial membrane depolarization (JC-1, green) and (**F**) superoxide production (Mitosox Red, red). DAPI staining for Cell nuclei (blue). (**G**) Analysis of flow cytometry for the apoptotic 4T1 cells induced by CEG upon 808 nm laser irradiation or not. Statistical differences were determined by Student’s t-test. * *p* < 0.05, ** *p* < 0.01, *** *p* < 0.001.

The noticeable photothermal and photodynamic performance inspires us to study the potential phototherapeutic effect when CEG acts as a PTT and PDT agent in murine mammary carcinoma (4T1) cells. Firstly, we assessed the biocompatibility of CEG (OD = 0, 0.25, 0.5, 1, and 2) using CCK-8 assay. Figure 4A displayed that the survival rate was more than 80% at the tested concentrations after 24 h incubation, indicating excellent biocompatibility. However, under 808 nm laser irradiation (1 W/cm^2^, 10 min), CEG showed significant cytotoxicity in a dose-dependent manner. Calcein acetoxymethyl (AM)/propidium iodide (PI) live/dead cell staining assay (Figure 4B) illustrated similar results with CCK-8 assay, further certifying the biocompatibility and phototherapeutic effect of CEG. Cellular hyperthermia and ROS production induced by PTT and PDT could induce the heat shock protein (HSP) [41,49] and phase II enzyme expression (typically HO-1) expression [31,50,51]. As a result, HSP-70 and HO-1 expression were detected via western blot. Figure 4C illustrated that CEG upregulated HSP-70 and HO-1 expression in a dose-dependent manner under 808 nm laser irradiation. In contrast, NIR alone or CEG without irradiation could not trigger HSP-70 or HO-1 expression. Overexpression of HSP-70 and HO-1 may induce the activation of oxidative stress-signaling pathways, leading to the generation of ROS and mitochondrial dysfunction [31,52]. The cellular ROS level was assessed by flow cytometry and confocal fluorescence microscopy (CLSM) using DCF assays. Figure 4D demonstrated that CEG could cause cellular ROS production under 808 nm irradiation, while there was no obvious ROS generation when cells were treated with NIR alone or CEG without NIR irradiation. Fluorescence microscopy images displayed similar results and further certified the dose-dependent DCF fluorescence enhancement of CEG upon 808 nm laser irradiation (Figure S7). Once cellular ROS generation exceeds the antioxidant defense capacity and fails to recover redox balance, mitochondrial dysfunction will occur, involving aberrant mitochondrial membrane depolarization and increased mitochondrial superoxide generation [50]. CLSM images showed that CEG triggered dose-dependent mitochondrial membrane potential depolarization (JC-1, green fluorescence) and superoxide production (Mitosox, red fluorescence) upon NIR irradiation (Figure 4E,F). In contrast, CEG or NIR alone could not cause mitochondrial dysfunction. Mitochondrial dysfunction-induced apoptosis was detected by Annexin V-FITC/PI apoptosis assay. As shown in Figure 4G, incubation of CEG for 24 h under NIR irradiation would cause 75.7% of early apoptosis and 20.1% of late apoptosis. In comparison, there was no noticeable 4T1 cell apoptosis CEG or NIR alone. In conclusion, a significant destructive effect was observed in CEG with the NIR irradiation group, which may ascribe to the activation of hierarchical oxidative stress induced by the PTT and PDT effects of CEG.

### 3.4. In Vivo Photoimmunotherapy of CEG Synergized with α-PD-1

**Figure 5:**
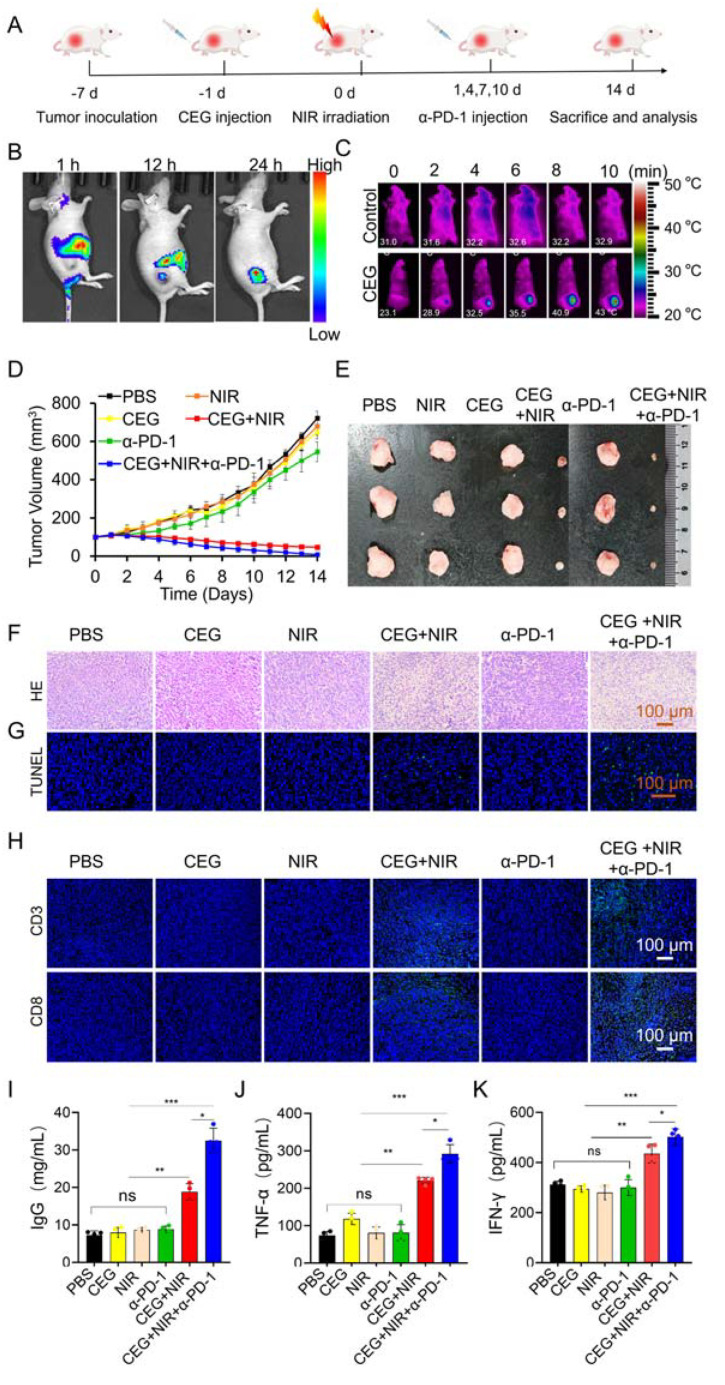
In vivo photoimmunotherapy of CEG synergized with α-PD-1 in 4T1 tumor-bearing mice. (**A**) Therapeutic protocol illustration of 4T1 tumor mice. (**B**) Fluorescence images of mice after intravenous injection of CEG-Cy7 at 1, 12, and 24 h. (**C**) Infrared thermal images of 4T1 tumor-bearing mice at 24 h post-injection of PBS or CEG with 808 nm laser radiation (1 W/cm^2^, 10 min). (**D**) Tumor growth curves of different groups for 14 days after intravenous injection of CEG. (**E**) Tumor images of different groups at the end of treatment. (**F**,**G**) H&E and TUNEL staining for the tumor tissues in each group. (**H**) Immunofluorescence staining for CD3 T cells and CD8 T cells in tumors after different treatments. (**I**) Serum IgG, (**J**) TNF-α, and (**K**) IFN-γ levels of six groups after different treatments measured by ELISA. Data are presented as means ± s.d. (*n* = 3). Statistical differences were determined by Student’s *t*-test. * *p* < 0.05, ** *p* < 0.01, *** *p* < 0.001.

Encouraged by the satisfactory phototherapeutic effects in vitro, in vivo photoimmunotherapy of CEG NRs under NIR laser (1 W/cm^2^) irradiation was assessed on 4T1 tumor-bearing mouse models. Figure 5A exhibits the animal experiment design. The tumor-bearing mice were randomly divided into six groups: (1) PBS, (2) CEG, (3) NIR, (4) CEG + NIR, (5) α-PD-1, and (6) CEG +NIR + α-PD-1. First, the biodistribution of CEG in vivo was tracked by fluorescence imaging of live animals. Cy7-labeled CEG (CEG-Cy7) was intravenously administered, and the fluorescence photographs were collected at 1, 12, and 24 h after administration. As shown in Figure 5B, there is a noticeable accumulation of CEG-Cy7 in the tumor site in a time-dependent manner as a result of the improved permeability and retention (EPR) effect. The abundant accumulation of CEG in the tumor region was a premise for the in vivo treatment. Then, the in vivo therapeutic effect of CEG was investigated after intravenous injection of CEG (OD = 60). As seen in the infrared thermal image (Figure 5C), the temperature of the tumor region raised from 26 °C to about 43 °C after intravenous administration of CEG (OD = 60) with NIR laser irradiation, promising for the PTT and PDT performance. Afterward, the anti-tumor effects of CEG were measured by monitoring the tumor growth for up to two weeks after treatment. The tumor growth profile (Figure 5D) and representative photographs of tumor tissues at the treatment endpoint (Figure 5E) revealed a remarkable tumor regression effect in the CEG + NIR and CEG + NIR + α-PD-1 groups, and the latter showed a better inhibition effect. In comparison, other groups demonstrated a little inhibitory effect on tumor growth. Then, harvested tumors of different groups were used for subsequent H&E staining and TUNEL staining. Figure 5F,G exhibited that CEG + NIR and CEG + NIR + α-PD-1 showed severe apoptosis as well as obvious tumor destruction under NIR laser irradiation, among which the latter had the best efficacy. In contrast, no significant damage was observed in tumor tissue in all of these groups without NIR light exposure.

In the process of photoimmunotherapy, cytotoxic T cells (CD8^+^ and CD3^+^ T cells) could trigger cell apoptosis by directly attacking cancer cells [53,54]. Consequently, CD8^+^ and CD3^+^ T cells in tumors were observed by immunofluorescence and immunohistochemical staining. Figure 5H and Figure S8 illustrated that the most significantly expanded CD8^+^ T cells and CD3^+^ T cells occurred in the CEG + NIR + α-PD-1 group, implying CEG + NIR + α-PD-1 could activate effective anti-tumor immune responses. The CEG + NIR group also promotes the infiltrations of CD8^+^ and CD3^+^ T cells, which may be ascribed to the fact that dying tumor cells after PTT and PDT can release TAAs, which act as an in situ cancer vaccine to activate the immune system against tumors. Since serum IgG was found to play a critical role in immune effector cells [55], we detected the serum IgG levels in mice among different groups. It was found that the CEG + NIR group could elevate the level of IgG, and the elevating effect was more pronounced after combining with α-PD-1 (Figure 5I). Meanwhile, serum TNF-α and IFN-γ levels were also analyzed due to the important role of TNF-α in triggering an anti-tumor immune response [56] as well as the crucial role of IFN-γ in intracellular immunity against cancer [57]. Figure 5J,K displayed that the CEG + NIR + α-PD-1 group exhibited the highest TNF-α and IFN-γ level, followed by the CEG + NIR group. Additionally, the synergistic effect of the CEG + NIR + α-PD-1 group was demonstrated by calculating the additive tumor inhibition ratio according to the following equation reported in the previous literature [58].

*T*_treatment_ = (1 − *f*_CEG + NIR_ × *f*_α-PD-1_) × 100% (1)

*f*_treatment_ = V_treatment_/V_control_ × 100% (2)

As shown in the equation, *f* represented the relative tumor growth rate after each treatment, and V represented the relative tumor volume. The calculation results showed that the tumor inhibition ratio measured for the CEG + NIR + α-PD-1 group was significantly higher than those of calculated values (additive group) beyond 10 days (Figure S10), indicating that the TAAs generation after photo destruction of tumors and α-PD-1 could cause the synergistic anti-tumor effect.

The biosafety of CEG was further evaluated by tracking the body weight and the pathological features of the checked organs. Figure S9 showed that the body weights increased steadily in all groups. Additionally, no obvious abnormalities or damage to the heart, liver, spleen, lung, or kidneys were found (Figure S11), indicating the favorable biocompatibility of CEG-based photoimmunotherapy.

## 4. Conclusions

In summary, we have synthesized CeO_2_ end-deposited gold nanorods (CEG) to achieve excellent NIR photoimmunotherapy. Compared with CBG NRs, the LSPR of CEG can be regulated to the NIR region, possessing excellent temperature evaluation controllability and ROS boost CBG under NIR light irradiation, resulting from the spatial distribution structure that makes hot electrons participate at the ends and hot holes be consumed at the exposed side surface, greatly enhancing the utilization efficiency of photocarriers, thus showing strong photothermal and photodynamic effects to destroy tumors and activate a part of the immune response. Simultaneously, the combination with a PD-1 antibody could reverse the immunosuppressive microenvironment and thoroughly activate the immune response by enhancing cytotoxic T lymphocyte infiltration. In conclusion, our research demonstrates the superiority of combination therapy of photoimmunotherapy and PD-1 blockade in TNBC therapy.

## Data Availability

Data will be made available from the corresponding author upon reasonable request.

## Supplementary Materials

The following supporting information can be downloaded at: https://www.mdpi.com/article/10.3390/pharmaceutics15041309/s1, Figure S1: EDX spectrum of CEG and CBG.; Figure S2: HRTEM image of CEG and CBG. Figure S3: Plot and linear fit of time versus negative natural logarithm of the temperature elevation for the cooling rate of GNRs, CEG, and CBG. Figure S4: Total ROS, ^•^OH, ^1^O_2,_ and O_2_^•−^ assessments on GNRs, CEG, and CBG without NIR laser irradiation by DCF (**a**), TA (**b**), SOSG (**c**) and superoxide anion assay (**d**), respectively. PBS was used as a control. Figure S5: Stability of CEG. Figure S6: Hydrodynamic size and zeta potential of CEG NRs in H_2_O and cell culture medium before and after PEGylation. Figure S7: Fluorescence microscopy images of 4T1 cells to detect intracellular ROS. Figure S8: Immunohistochemistry staining for CD8 and CD3 T cells in tumor tissues after various treatments indicated. Figure S9: Body weight of mice from different groups for 14 days after various treatments. Figure S10: Tumor inhibition ratio of the CEG + NIR + α-PD-1 group and additive tumor inhibition ratio of combination therapy of photoimmunotherapy and PD-1 blockade. Figure S11: H&E staining histological images of heart, liver, spleen, lung, and kidney collected at the end of treatment. Tables S1 and S2: The wt% and At % values of all elements in.
