# Supplementary material for: Cerium End-Deposited Gold Nanorods-Based Photoimmunotherapy for Boosting Tumor Immunogenicity
Source: Pharmaceutics. 2023 Apr 21;15(4):1309. doi: 10.3390/pharmaceutics15041309 (PMC10145050; doi:10.3390/pharmaceutics15041309)
Supplement: Supplementary file 1 [file pharmaceutics-15-01309-s001.zip › pharmaceutics-2265834-supplementary.pdf]

Supplementary materials

Cerium End-deposited Gold Nanorods-based Photoimmunotherapy for Boosting Tumor Immunogenicity

Yanlin Feng [1,‡], Yumei Xu [2,‡], Zhaoyang Wen [1], Xin Ning [1], Jianlin Wang [1], Deping Wang [1,*], Jimin Cao [1], Xin Zhou [1,2,*]

[1] Key Laboratory of Cellular Physiology, Ministry of Education, and the Department of Physiology, Shanxi Medical University, Taiyuan 030001, China

[2] Department of Medical Imaging, Shanxi Medical University, Taiyuan 030001, China

* Correspondence: wangdeping@sxmu.edu.cn (D. Wang), xzhou@sxmu.edu.cn (X. Zhou)

\# These authors contributed equally to this work.

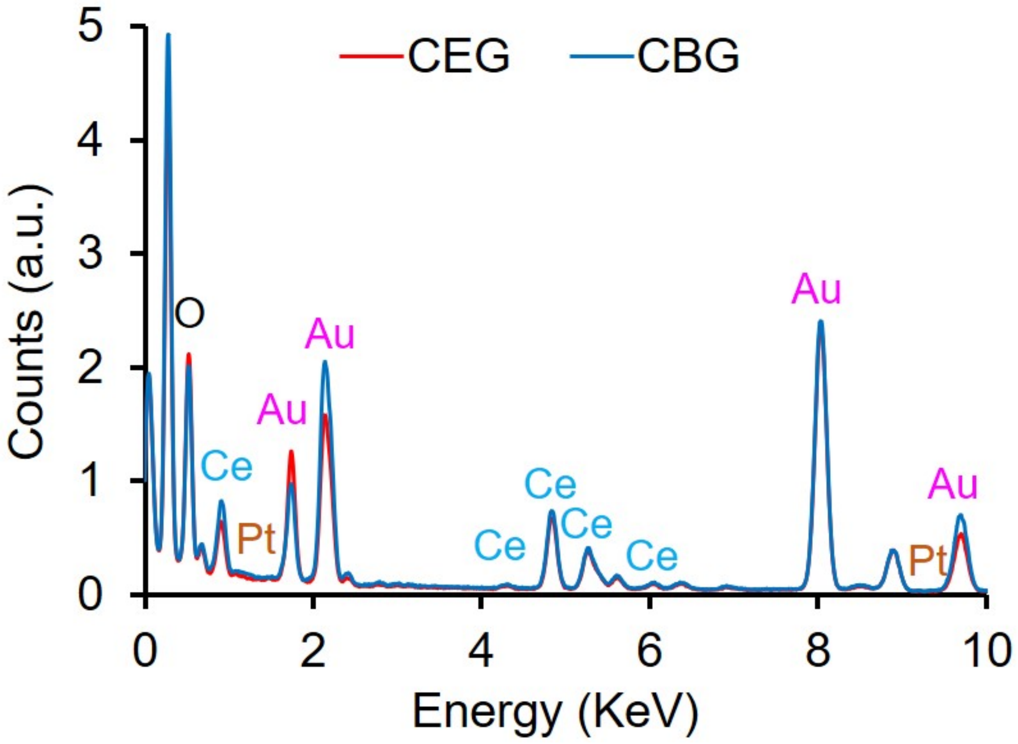

Figure S1 EDX spectrum of CEG and CBG.

The wt% and At % values of all elements in.

Element	wt%	At%
O	21.27	74.22
Ce	30.16	12.02
Pt	2.04	0.58
Au	46.53	13.19

Element	wt%	At%
O	25.43	78.17
Ce	31.73	11.13
Pt	2.78	0.70
Au	40.06	10.00

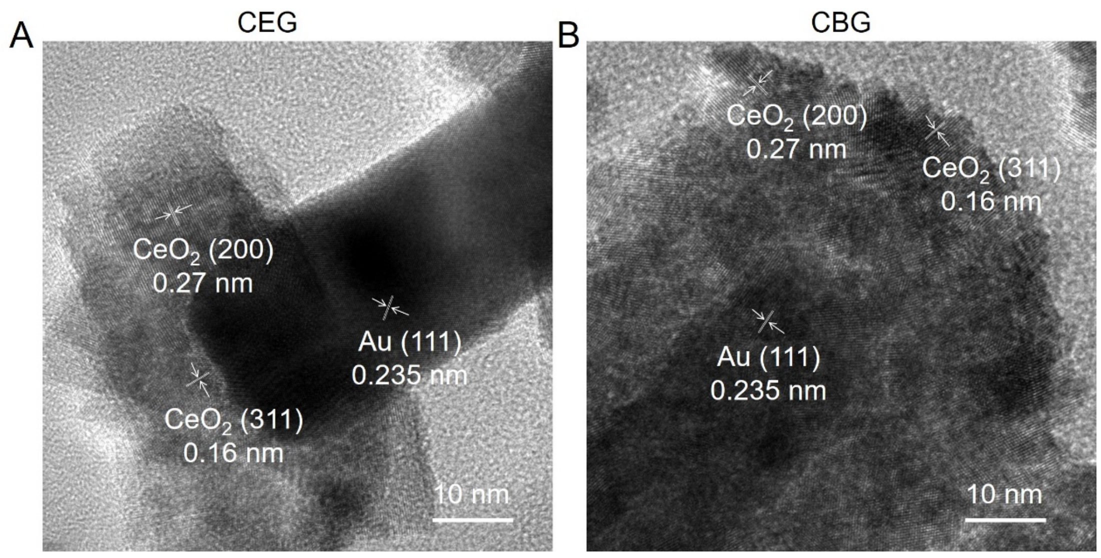

Figure S2 HRTEM image of CEG and CBG.

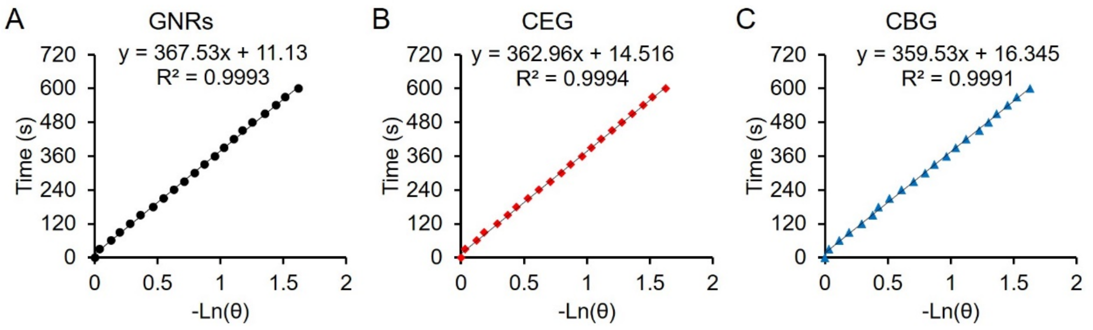

Figure S3 Plot and linear fit of time versus negative natural logarithm of the temperature elevation for the cooling rate of GNRs, CEG and CBG.

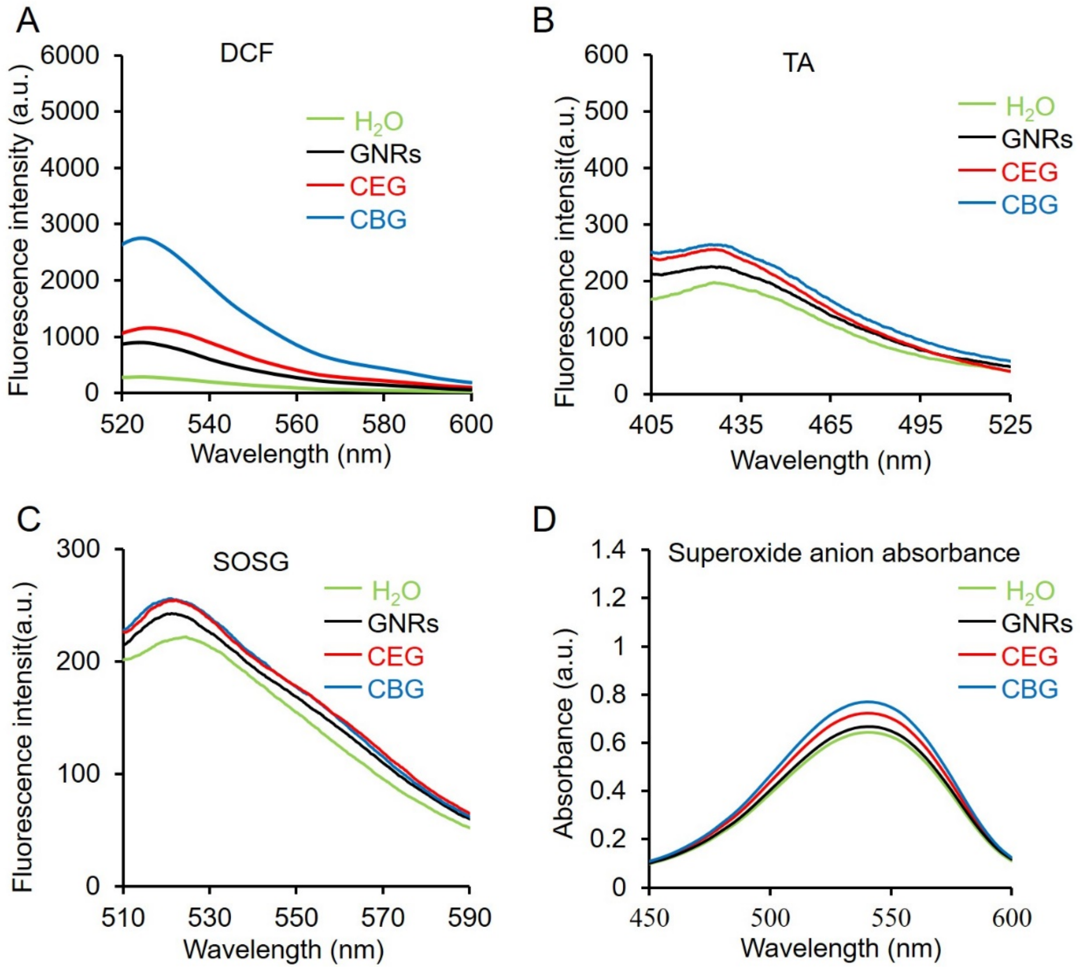

Figure S4 Total ROS, •OH, $^1O_2$ and $O_2•^-$ assessments on GNRs, CEG and CBG without NIR laser irradiation by DCF (a), TA (b), SOSG (c) and superoxide anion assay (d), respectively. PBS was used as control.

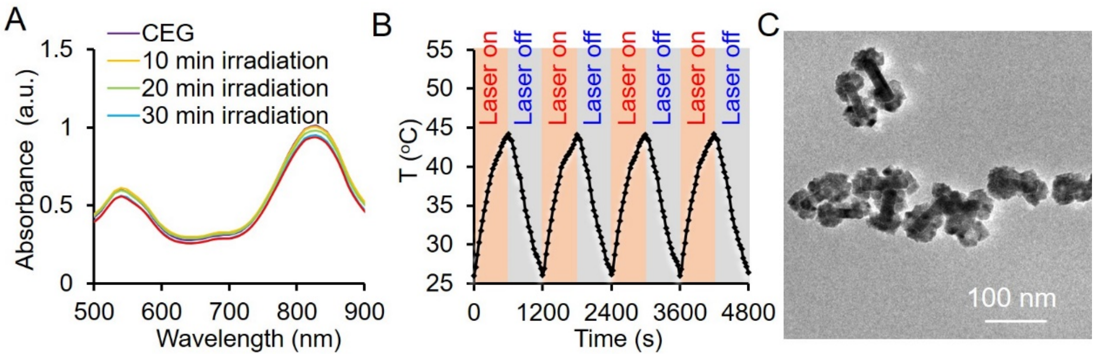

Figure S5 Stability of CEG. (A) UV-Vis spectra of CEG before and after NIR irradiation. (B) The photothermal profiles of CEG (OD=1 over 4 irradiation cycles. (C) TEM image of CEG after 4 irradiation cycles.

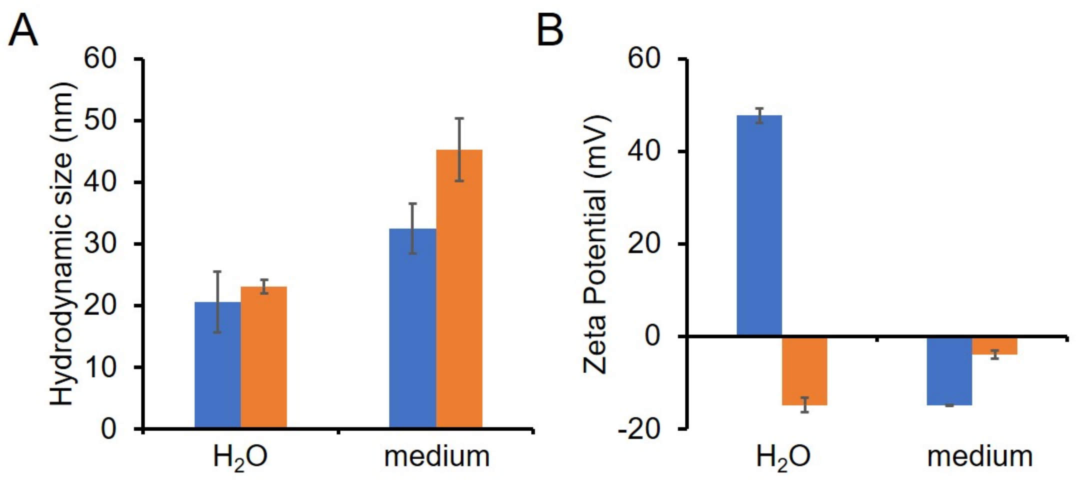

Figure S6 (A) Hydrodynamic size and (B) Zeta potential of CEG NRs in H_2O and cell culture medium before and after PEGylation.

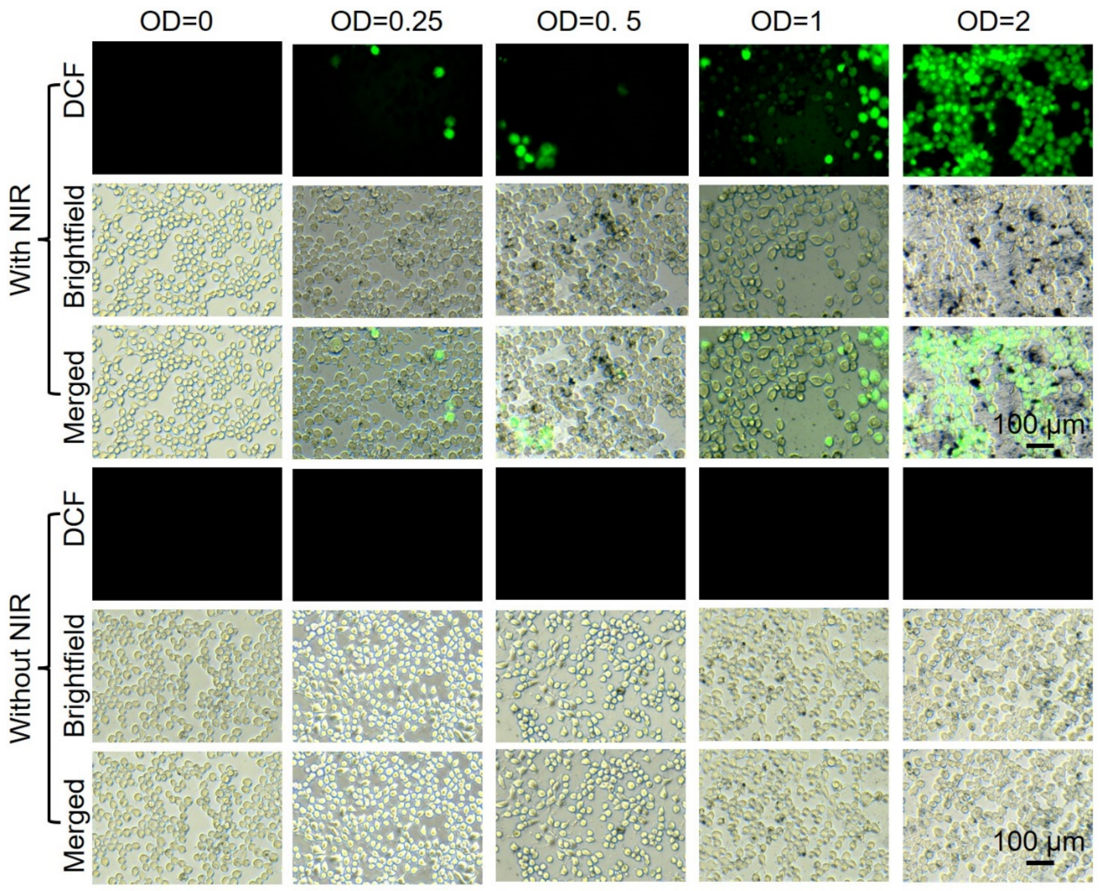

Figure S7 Fluorescence microscopy images of 4T1 cells to detect intracellular ROS (DCF, green).

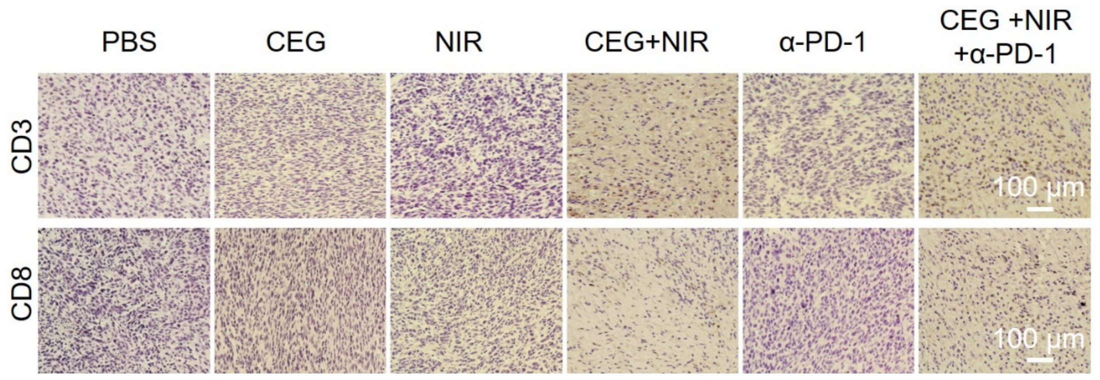

Figure S8 Immunohistochemistry staining for CD8 and CD3 T cells in tumor tissues after various treatments indicated.

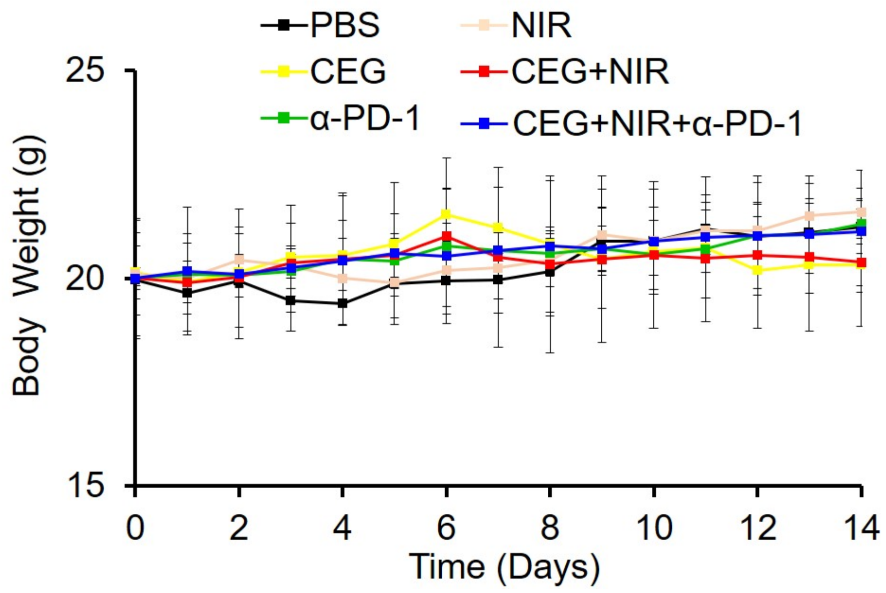

Figure S9 Body weight of mice from different groups for 14 days after various treatments.

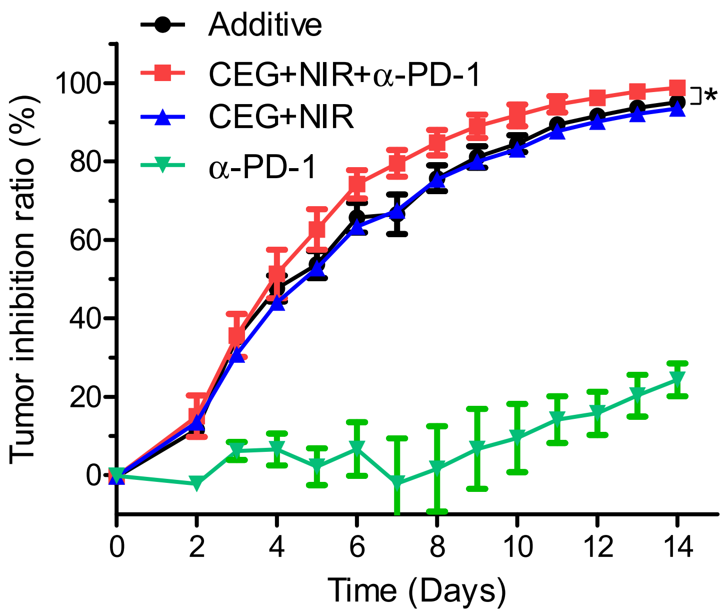

Figure S10 Tumor inhibition ratio of the CEG+NIR+α-PD-1 group and additive tumor inhibition ratio of combination therapy of photoimmunotherapy and PD-1 blockade.

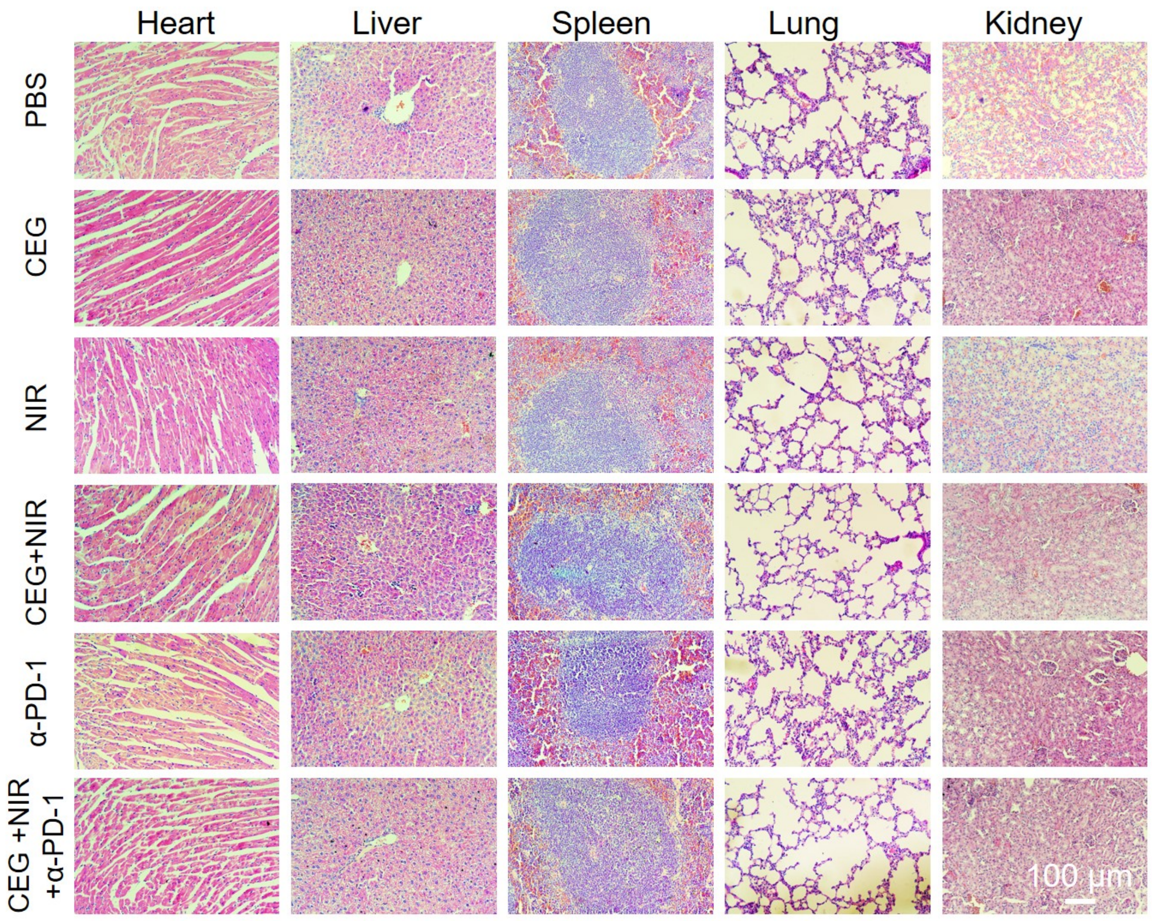

Figure S11 H&E staining histological images of heart, liver, spleen, lung, and kidney collected at the end of treatment.
